# Analytic Framework to Determine Proximity in Relationship Coffee Models

**DOI:** 10.1111/soru.12278

**Published:** 2019-11-17

**Authors:** Hanna Edelmann, Xiomara Fernanda Quiñones‐Ruiz, Marianne Penker

**Affiliations:** ^1^ Institute for Sustainable Economic Development Department of Economics and Social Sciences University of Natural Resources and Life Sciences Vienna (BOKU), Feistmantelstraße 4 1180 Vienna Austria

## Abstract

In conventional food systems, there are often large social and geographical distances between production and consumption. Alternative food networks (AFNs) like relationship coffee models aim to shorten these distances through direct contacts, communication, trust, transparency or commitment to improve farmers’ livelihoods. These relationship coffee models appear in diverse shapes with various implications for producers. Therefore, we deductively develop a framework to conceptualise proximity in four dimensions (organisational, institutional, cognitive and social) with subdimensions and three transversal dimensions ((temporary) geographical proximity, power, and communication). The analytic framework is complemented by an illustrative case to empirically test it, showing high geographical, organisational, institutional and cognitive proximity but low social proximity between coffee producer and restaurant owner. For future research, the framework can help to conceptualise proximity or to distinguish different types of relationship coffee models and to unpack conditions under which relationship coffees can increase proximity between coffee producers and buyers, often located far apart.

## Introduction

Conventional food systems have become industrialised and globalised, often leading to a vast geographical and social distance between consumers and producers (Krausmann and Langthaler 2019). Alternative food networks (AFNs) are trying to bridge this distance and re‐embed food into their socio‐ecological production context through direct and personal ties between producers and consumers (Hinrichs 2000).

In AFN literature, reduced distance does not only mean that there is less geographical space between producers and consumers, or no/few intermediaries (e.g., Kebir and Torre 2013; Kneafsey *et al.* 2013; Thorsøe and Kjeldsen 2016). Definitions of AFNs generally include small‐scale producers using organic or holistic production methods, meeting and purchasing places like farmers’ markets, and social/environmental/economic concerns for sustainability in production, distribution, and consumption (Jarosz 2008). Bos and Owen (2016) researched virtual communication for reconnecting producers and consumers in AFNs, concluding that they cannot fully replace personal experiences, but call for further research. Dubois (2018) enriches AFN literature by connecting it with the theoretical concept of proximity (Torre and Gilly 2000; Boschma 2005) and shows how geographical, cognitive, organisational, institutional and social proximity in AFNs is established and developed.

Conventional coffee value chains normally show little proximity: usually, coffee is produced in the global South and traded via various intermediaries to the global North, with little or no direct interactions between roasters, consumers and coffee producers (Daviron and Ponte 2005). Buyers and roasters act as gatekeepers for information exchange between producers and consumers (Borrella *et al.* 2015). Challenges in the coffee sector include market concentration, volatile farm gate prices, poor living conditions of producers and environmental degradation (Tröster and Staritz 2015; Samper and Quiñones‐Ruiz 2017; Panhuysen and Pierrot 2018). Certifications (e.g., Organic, Fairtrade, Rainforest Alliance) were designed to overcome these challenges, but criticism towards their effectiveness exists (Muradian and Pelupessy 2005; FAO 2014; Dietz *et al.* 2018).

Over the past years, direct trade and relationship coffee emerged, intending to go further/to do better than conventional coffee value chains and voluntary standards. Vicol *et al. *(2018, p. 27) define relationship coffee as

‘coffee marketed to consumers as being procured through a direct relationship between roaster and producer typically involving personal interaction, mutual trust, price transparency, a commitment to quality improvement and (importantly) a stated intention to improve the lives of coffee farmers and their communities’.

Like in other types of AFNs, there is supposedly some form of proximity between chain actors, despite the geographical distance of production and consumption (Vicol *et al.* 2018). However, over time, the term direct trade has been co‐opted by an increasing number of coffee roasters (MacGregor *et al.* 2017). The first direct trading companies are currently drawing back from the term because other roasters are extensively using it in their marketing strategy without complying with the initially high moral standards (MacGregor *et al.* 2017). Among Fairtrade buyers, Raynolds (2009) differentiated between mission‐driven, quality‐driven and market‐driven buyers with trade norms ranging from partnership to mere traceability. While mission‐driven buyers strongly advocate Fairtrade’s principles, quality‐driven buyers use Fairtrade as a mechanism to source excellent coffees, and market‐driven buyers comply with certification standards, but other than that continue mainstream business‐as‐usual (Raynolds 2009). We assume that a similar differentiation of trade models is valid for relationship coffees leading to different implications for coffee producers. To compare these, it is crucial to understand in more detail how relationship coffee models work and the differences among them. Following Vicol *et al. *(2018), relationship coffees claim to have more closeness between the actors while being geographically very long. However, there seem to be variances in the proximity among actors. Therefore, the factors that make up this proximity need to be further investigated to identify the conditions under which relationship coffees can help producers to cope with the prevailing challenges in the coffee sector.

Thus, the main purpose of this publication is to develop a framework that conceptualises dimensions of proximity in coffee value chains. The framework aims to contribute to the debate on how to design best practices/guidelines along the specialty coffee value chain (see e.g., Panhuysen and Pierrot 2018; Transparent Trade Coffee 2018). Furthermore, to illustrate the framework’s applicability, we want to give insights into an exemplary relationship coffee model to empirically test the proposed proximity dimensions. The corresponding research question is: how can proximity in relationship coffee models be conceptualised? The analytic proximity framework, presented in this article, was developed and tested to support empirical studies on proximity in coffee and other agro‐food chains. The next section of the article gives an overview of the literature on relationship coffee (including literature on third wave coffee, direct trade coffee, and sustainable sourcing programmes). The following section conceptualises the dimensions of proximity for relationship coffee models, followed by the method section. After this, we test the analytic proximity framework by analysing a case study, followed by a discussion and conclusion.

## Relationship coffee models in the third wave of coffee

Relationship coffees are generally linked to the so‐called third wave of coffee. The first wave started in the 1930s, where mass production and consumption were dominant. The second wave was driven by the US‐American roasters Peet’s and Starbucks and the coffee sector became more differentiated by quality. In the third wave, coffee is sold as an artisanal product, differentiated by quality, origin, specialty roasting, preparation, or some form of social and/or environmental sustainability promise (Borrella *et al.* 2015; Boaventura *et al.* 2018). It is important to notice that these developments occur in the world of specialty coffee. Specialty coffee is distinguished from industrial coffee through its high quality, restricted supply, freshness, special flavours, package, or consumption atmosphere (Bacon 2005; Daviron and Ponte 2005). The protocol of the Specialty Coffee Association of America and Europe (SCA) helps to determine the organoleptic quality of a coffee. In so‐called cuppings trained experts assess the cup quality on a scale up to 100 points. Specialty coffee has to score more than 80 points, and third wave coffee generally even higher (Fischer 2017). Relationship coffee models might include some intermediary parties as long as there is a direct relationship between roaster and producer (Vicol *et al.* 2018, see definition above). Generally, there is some form of direct interaction to coordinate quality and communication (Holland *et al.* 2016). Roasters often argue that through their relationship coffee model, they are avoiding bureaucratic and ineffective voluntary standards (Vicol *et al.* 2018). Panhuysen and Pierrot (2018, p. 21) define these relationship‐based sourcing programs as

‘a known relationship with producers that goes beyond the transactional to include a sense of equity manifest in mutual and transparent processes that promote best practices in coffee production and processing, to safeguard the rights and well‐being of producers, workers, the community and the environment’.

Thus, they stress the principles of transparency, equality, participation and a commitment to enhancing social and environmental production conditions.

We are considering relationship coffees as chains that show a direct interaction between producers and buyer/roaster who sells the coffee to the final consumers. This definition explicitly excludes value chains that include a sourcing company even if they provide transparent information about the producers to the roasters, but do not facilitate immediate contacts or further interactions. In other words, we are focusing on value chains where roasters and producers know and directly talk to each other, instead of only relying on what Borrella *et al. *(2015) call connective businesses linking producers and specialty roasters more directly than conventional traders. Borrella *et al. *(2015) show that they bring economic benefits for both parties and that they empower producers. Trust and commitment of both roasters and producers are crucial. MacGregor *et al. *(2017) studied direct trade of coffee in the USA and Scandinavia by looking at the founding direct trade roasters. They found that direct trading started as a tool to improve coffee quality and producers’ income and to establish regular communication. However, the initial direct traders are struggling with other coffee companies’ efforts to use the term mainly for marketing but not acting after the core principles of direct trade (MacGregor *et al.* 2017). Fischer (2017) studied value creation in the third wave specialty coffee market and concluded that coffee producers often lack the social capital to benefit substantially from the higher value of top‐quality specialty coffee. In a study of a tasting encounter between Danish coffee roasters and Colombian coffee exporters, Holland *et al. *(2016) observed that coffee quality is not given nor static but rather constructed in negotiations between the actors. The growing third wave coffee shops are emphasising their specific blending and roasting methods and organoleptic qualities of the cup instead of highlighting the coffee origin and local production. Thus, the benefits of increased consumer awareness and the resulting willingness to pay a higher price go rather to the roasters/coffee shop owners than to producers (Fischer 2017). Boaventura *et al. *(2018) argue that closer collaboration of producers with upstream value chain actors could conquer this difficulty. To establish close relationships along the chain, it helps when value chain actors have advanced knowledge of each other’s practices and products. In a recent and extensive study on relationship coffee models in Indonesia, Vicol *et al. *(2018) concluded that in their case studies, the trading model rather reproduced local inequalities and favoured local elites rather than contributing to rural development.

Following these considerations, we argue that relationship coffees could be seen as a form of AFNs. Bos and Owen (2016) show that AFNs can also be facilitated through virtual connections between producers and consumers, and partially compensate for lack of geographical proximity. So, apart from the geographical dimension, what other dimensions of proximity are present in coffee value chains, and how can they be determined? Therefore, the next section is going to develop a framework to conceptualise proximity for coffee value chains, drawing on literature from AFNs and proximity scholars.

## Conceptualising proximity in global value chains

Polanyi (2001 [1944]) laid the basis for the embeddedness‐discourse by arguing that economic interactions in pre‐capitalist societies were embedded in social relations, politics, and religion. Emerging self‐regulating markets reduced the (social) mechanisms based on trust and reciprocal understanding for the sake of the price mechanism, and disembed markets from society. Granovetter (1985) opposes and states that economic transactions are always socially embedded. Decisions are not only taken because of the economic goals but also because of ‘sociability, approval, status and power’ (Granovetter 1985, p. 506).

Thus, compared to the conventional food system, AFNs often follow the goal to re‐embed food products more strongly in their social production contexts by shortening the distance between production and consumption through short value chains and/or by transmitting notions of community, care or stewardship (Marsden *et al.* 2000; Goodman and DuPuis 2002; Renting *et al.* 2003; Venn *et al.* 2006; Bowen and Mutersbaugh 2014). Social embeddedness is established through personal trust, reciprocity, solidarity, and familiarity (Hinrichs 2000; Thorsøe and Kjeldsen 2016; Dubois 2018). Moreover, AFNs are generally based on a different definition of quality (e.g., grounded in origin, traditional methods, taste, and environmentally friendly production) (Murdoch *et al.* 2000; Renting *et al.* 2003).

Proximity scholars have been looking into the relationships of actors and how other forms of proximity can substitute or complement geographical proximity (Torre and Gilly 2000; Torre and Rallet 2005). Apart from geographical proximity, Boschma (2005) distinguishes between cognitive proximity (actors have an overlapping knowledge base, essential for communication and learning), social proximity (based on trust through friendship, familiarity or kinship), organisational proximity (relations through organisational arrangements), and institutional proximity (actors share common norms, rules, values). Coming from France, a discourse on proximities in agriculture emerges and slowly feeds into international AFN literature (Filippi *et al*. 2011; Aubry and Kebir 2013; Kebir and Torre 2013; Dubois 2018). But to our knowledge, no scientific study has looked at coffee value chains through the lens of proximity. Moreover, we are still missing a solid conceptualisation of the proximity concept for global food value chains. Thus, drawing on proximity literature, we develop a framework to analyse proximities in relationship coffee models and to make their differences visible. The next section elaborates the framework in more detail, and Table 1 gives an overview of the proposed proximity dimensions in relationship coffee models, including coffee producers, buyers, and roasters.

**Table 1:** Proximity dimensions in relationship coffee models (based on literature presented above)

Proximity dimensions (based on Boschma, 2005)	Subdimensions	Range			
Organisational proximity – relations established through organisational arrangements	Organisational agreements	Number/range of areas regulated, formal/informal	**(Temporary) geographical proximity** (short‐term, personal meetings)	**Power** (direct or diffuse, dyadic or collective)	**Communication** (direct, indirect, frequency)
	Mode of price setting/negotiation	prices set by buyers – negotiation process			
	Duration of trade relations	On demand/short‐term – regularly/long‐term			
	Benefit sharing	One actor takes all benefits – returns are equally shared			
	Risk sharing	One actor takes all risk – risk is equally shared			
Institutional proximity – actors share common quality norms, rules, values	Coffee quality norms/ rules/ conventions	Different – same understanding of coffee quality			
	Certifications (e.g. Organic, Fairtrade)	Different – same importance for actors			
	Environmental norms (beyond certification)	Different – same importance for actors			
	Social practices (excl. payment)	Different – same importance for actors			
Cognitive proximity – actors have an overlapping knowledge base	Knowledge bases of actors (production, processing, preparation, consumption, quality)	Different – same level and type of knowledge			
	Knowledge exchange, learning	None – mutual learning mechanisms			
Social proximity – relations based on trust through friendship, familiarity or kinship	Trust	None – totally trustful relations			
	Knowledge about each other	No knowledge about actors – strong interpersonal ties			
	Degree of personal acquaintance	No relationship – friendship, kinship			

### Organisational proximity

According to Boschma (2005, p. 65), organisational proximity refers to the ‘extent to which relations are shared in an organizational arrangement, either within or between organizations. To be precise, this involves the rate of autonomy and degree of control that can be exerted in organizational arrangements’. It ranges from no ties between actors (e.g., spot market) over loosely coupled networks (joint venture or loose firm network) to strong ties (highly organised firm or network) (Boschma 2005). Dubois (2018) also includes the practices that actors use to manage their flows of information, money, and food. In conventional coffee value chains, producers are often selling coffee beans to local buyers or cooperatives without any ties to upstream value chain actors (Daviron and Ponte 2005; Jaffee 2007). Moreover, producers bear most of the price instabilities and risks (Bargawi and Newman 2013; Tröster and Staritz 2015). To tackle this challenge, some relationship coffee models address the issue by establishing long‐term contracts (MacGregor *et al.* 2017). In the definition of sustainable sourcing, Panhuysen and Pierrot (2018) emphasise the role of equity and producer participation for a balanced trade relationship. Thus, our framework analyses the structure of the value chain:

- Organisational agreements: what areas of the relationship are regulated (price, logistics, financing), and how (e.g., via formal contracts, or only verbally/informal)?
- Mode of price setting/negotiation: how is the price determined (only by the buyers or through negotiations with producers)?
- Duration of trade relation: in what timeframe do the actors cooperate? (on‐demand/short‐term or regularly/long‐term)
- Benefit sharing: how are the returns distributed? (only based on current market prices, or are higher returns equally shared among actors)
- Risk sharing: who is taking the risk in the trade?

Following this, low organisational proximity in a coffee value chain would mean that there are no ties between producers and buyers and they only interact once or infrequently to sell and buy coffee. The other end of the continuum would be a hierarchical network (of growers, processors, roasters and retailers) with strongly regulated interactions or one single firm that grows, processes, roasts and sells the coffee (Boschma 2005). Organisational proximity can be fostered through direct and regular communication (Dubois 2018) or more formally through firm integration via, e.g., mergers and acquisitions (Balland *et al.* 2015).

For better illustration of the organisational proximity, Table 2 depicts the actors of three exemplary relationship coffee models. Albeit our definition of relationship coffee requires direct interactions, relationship coffees can also include other actors facilitating the trade (e.g. export/import, logistics, storage) while still encouraging direct contacts (Borrella *et al.* 2015). Sole service providers (e.g., a shipping company that transports the beans from the port of origin to the port of destination) are not shown.

**Table 2:** Examples of relationship coffee models

Relationship coffee models	Value chain and corresponding activities	What is happening?
Producers are organised in a cooperative and sell their green coffee beans to a roaster.	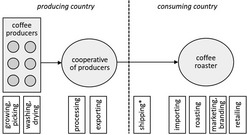	Coffee producers are organised in a cooperative. Individual roasters buy coffee directly from the producer co‐operative and organise the import.
Individual producer sells to a roaster.	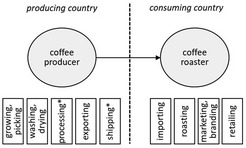	Individual coffee producer sells coffee beans to a roaster, coffee producer organises the export.
Trading platform facilitates trade and establishes direct contacts.	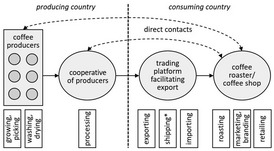	Coffee producers are organised in a co‐operative. Intermediaries facilitate export while establishing direct contacts between roasters and producers.

Note:

*Activity done by service provider, coffee does not change the owner. Black arrows indicate the flow of the coffee beans.

### Institutional proximity

Institutional proximity covers the closeness of actors’ institutions. Institutions in this context are formal (e.g., laws, regulations) or informal (e.g., customs, traditions, taboos, norms) rules that structure human interactions (North 1991). If actors share the same or similar institutions – like the same language, common values, similar habits, and common laws and rules – it fosters information sharing, economic coordination and interactive learning (Boschma 2005). Balland *et al. *(2015) argue that through institutionalisation processes during frequent cooperation, actors incorporate rules, values or ethical principles into their behaviours. Mutual experiences dealing with food origin and quality can lead to a conveyance of the values (Dubois 2018). As a result, actors sometimes codify the institutions in a guideline for cooperation (Balland *et al.* 2015). Therefore, the framework highlights the important institutions and analyses if they are the same or different for value chain actors.

Relationship coffees often emphasise the beans’ quality (Fischer 2017). Quality highly impacts farm gate prices (Transparent Trade Coffee 2018). Quality is a result of construction and negotiation processes (Holland *et al.* 2016) and acts as in‐ or excluding market mechanism (Fischer 2017). Therefore, it is important to understand how coffee quality is defined and if these definitions align among the actors. Further quality institutions can be certifications (e.g., Organic, Fairtrade, Rainforest Alliance) that the actors adhere to and value (emotionally or monetary) (e.g., Muradian and Pelupessy 2005; Raynolds *et al.* 2007). Environmental norms (beyond certifications) can be upheld and important to the actors (Panhuysen and Pierrot 2018), as well as other social or ethical principles (e.g., community support, development projects implemented by buyers) (MacGregor *et al.* 2017; Panhuysen and Pierrot 2018). Institutional proximity can be developed through common experiences, supported by the transmission of value‐laden information and depend on the actors’ agenda, motivation for actions and benevolence (Kebir and Torre 2013; Dubois 2018).

Thus the framework analyses the importance of following quality institutions (understood as norms, rules, or practices) in the value chain:

- Coffee quality norms/rules/conventions,
- Certifications,
- Environmental norms (beyond certification),
- Social practices (excluding payment).

Following these considerations, a scale of institutional proximity would range from very different to exactly the same quality institutions among the actors in the value chain.

### Cognitive proximity

Cognitive proximity is the convergence of the actors’ knowledge bases. Knowledge is not entirely and easily transferable because of its cumulative, localised and tacit quality. For mutual communication, understanding, and processing of information, the knowledge bases of the actors should at least partly overlap (Boschma 2005). Learning processes can reduce cognitive proximity and if there are constant interactions, most likely, the knowledge bases will become more similar over time (Balland *et al.* 2015). However, innovations and learning from others can benefit from heterogeneous knowledge bases (less cognitive proximity) (Balland *et al.* 2015).

The coffee literature (i.e., Boaventura *et al.* 2018) explains that an increase of farmers’ expertise in coffee production, processing, preparation, consumption and consumers’ quality perceptions improves the quality of the product, farmers’ bargaining position and following this, their income. Vice versa, the education of buyers and consumers about coffee, production conditions and the producers themselves is important to create mutual understanding (Boaventura *et al.* 2018). Therefore, our framework considers the dimensions:

- Differences and similarities of actors’ knowledge bases (production, processing, preparation, consumption),
- Forms of knowledge exchange and learning.

In other words, high cognitive proximity would mean that actors have the same knowledge concerning coffee production/preparation/consumption and that opportunities for substantial knowledge exchange or even mutual learning mechanisms (e.g., workshops, training) occur.

Cognitive proximity can increase through interpersonal relations (Dubois 2018) fostering learning from each other (Balland *et al.* 2015). Therefore, frequent and direct communication on production and business practices can encourage mutual learning – which often is an underlying principle of relationship coffees (MacGregor *et al.* 2017; Rosenberg *et al.* 2018; Vicol *et al.* 2018) – and thus foster cognitive proximity.

### Social proximity

Social proximity ‘occurs in the form of friendship or kinship or also based on past interactions. More than any of the other dimensions of proximity, social proximity relies on trust and in this manner encourages actors to engage in communication’ (Mattes 2012, p. 1089 drawing on Boschma 2005). Higher degrees of the personal acquaintance of actors (Balland *et al.* 2015) encourage open communication and potentially reduce opportunistic behaviour, with trust as a fundamental basis (Boschma 2005). Proximity dynamics become particularly visible in decoupling processes when relationships that are taken out of their usual contexts continue to exist (e.g., former co‐workers staying friends) (Balland *et al.* 2015).

In the definition of trust, we follow Saunders *et al. *(2014, p. 640) who draw on further authors (Mayer *et al.* 1995; Lewicki *et al.* 1998; Rousseau *et al.* 1998) stating ‘trust is depicted as occurring under conditions of risk that require the trusting party (the “trustor”) to develop favourable expectations of the intentions and behavior of the other party (“trustee”), sufficient to prompt a willingness to become vulnerable to the trustee’s future conduct’. Mayer *et al. *(1995) explain that trust is constituted by the trustor’s willingness to trust in general, and the trustworthiness of the trustee. Trustworthiness can emerge from trustee’s ability (skills or competencies, often in a specific task or field), benevolence (positive personal orientation/motivation/intention towards the trustor) and integrity (compliance to principles that are accepted by the trustor, e.g., justice, credibility, consistency of actions, depending on what is important for the trustor). They also argue that taking actions based on trust means taking a risk that leads to an outcome (Mayer *et al.* 1995).

Many studies on relationship coffees state that the relationship between the actors potentially involves some form of trust (Borrella *et al.* 2015; MacGregor *et al.* 2017; Boaventura *et al.* 2018; Rosenberg *et al.* 2018; Vicol *et al.* 2018). Borrella *et al. *(2015) explain that for the participants in their study, mutual commitment and trust were important to overcome risks of opportunistic behaviour. Vicol *et al. *(2018) show that disruption of trust can lead to a breakdown of business relations. Therefore, we aim to assess social proximity by looking at these three dimensions:

- Trust,
- Knowledge about each other,
- Degree of personal acquaintance.

Therefore, high social proximity would mean that the value chain actors are more than trading partners, maybe even friends and that they know each other very well. Social proximity can be established and developed through trust building via integrity, reputation and reliability, previous experiences and a mutual, common motivation, agenda or benevolence of actors (Balland *et al.* 2015; Dubois 2018; Ayari and Zaibet 2019).

It is important to notice that the four dimensions of proximity (see above) are very interconnected and overlapping. For example, social proximity can reduce cognitive distance after some time as it encourages mutual learning. Organisational proximity can compensate for low social proximity (lack of trust), and (temporary) geographical proximity can nurture social proximity through personal interactions and trust development (Boschma 2005).

Too much proximity can also have detrimental effects. It can increase the opportunities of malfeasance, force, and fraud (Granovetter 1985). Actors might miss out on new opportunities and stay locked in their present way of handling things (Uzzi 1997). Moreover, strong proximities could also exclude new actors, ideas, and innovation or may create dependencies (Boschma 2005). Apart from these four dimensions of proximity, our framework also contains three transversal dimensions that are influencing each dimension and the concerning sub‐dimensions (see Table 1).

### (Temporary) geographical proximity

Coffee value chains are traditionally very long, and thus there is often no permanent geographical proximity between the actors. However, Torre (2008) states that temporary geographical proximity can be established through short‐ or medium‐term face‐to‐face visits at crucial stages. These short‐term meetings can be sufficient to exchange, communicate and build up other forms of proximities for co‐operation, making permanent geographical co‐location of firms not a precondition for knowledge transfers. Fares, trade shows or conferences can be places where actors meet and exchange, but also individual face‐to‐face meetings and visits in specific stages of the cooperation: In the beginning of a cooperation to get to know each other and to set the rules, and then more or less regular visits to solve problems or conflicts and to plan the further cooperation (Torre 2008). Virtual communication between the meetings helps to bridge the time between face‐to‐face meetings (Bos and Owen 2016). Relationship coffee models are often based on personal and regular contacts of coffee buyers (Vicol *et al.* 2018). MacGregor *et al. *(2017) show that all six direct trade coffee companies which they studied, committed to regular producer visits. In our framework, we look at how often and how regularly producers and buyers meet and how that influences other proximity dimensions.

### Power

Our framework combines the proximity dimensions but also adds another transversal layer concerning power. Muradian and Pelupessy (2005, p. 2030) define value chain governance as the ‘extent to which the leading segment(s) exert control over information exchange and production activities, and therefore are able to shape the functional division of labor along the chain and to set entry barriers’. Therefore, it is necessary to assess not only the proximity of value chain actors but also the inherent power relations – who is taking the initiative and who is adapting. Conventional coffee value chains are considered as buyer‐driven (Ponte 2002; Raynolds 2009). That means that large retailers set the standards of the trade relationships that producers have to comply with (Gereffi 1994). Relationship coffee models tend to emphasise mutuality and equity among producers and buyers (Panhuysen and Pierrot 2018; Vicol *et al.* 2018). However, Dallas *et al*. (2017) argue that all business relationships show power differentials. Some governance models might have stronger power imbalances than others, and this is not necessarily negative. They differentiate between direct and diffuse forms of power transmission: direct transmission refers to intentional power exertion of actors with clear aims, positions or resources. Diffuse transmission is less explicit, can be unintentional and occurs in the forms of societal norms, quality conventions or e.g., best‐practice examples. Dallas *et al*. (2017) also differentiate between dyadic and collective arenas of actors, dyadic referring to two parties (individuals or groups) involved in a power relationship, while in collective arenas collectives (e.g., co‐operatives, multi‐stakeholder initiatives, and associations) hold and exert power. The authors identify four types of power: bargaining power (direct transmission, dyadic actors), institutional power (direct transmission, collective actors), demonstrative power (diffuse transmission, dyadic actors) and constitutive power (diffuse transmission, collective actors) (Dallas *et al*. 2017). The use of this typology helps us to further understand the variances in power relations. Grabs and Ponte (2019) show that in the differentiated specialty coffee sector, large companies use their demonstrative power ‘privatising the definition of quality and sustainability’ (Grabs and Ponte 2019, p. 19) and thus impose these definitions to producing countries. Getting insights into the mutuality, equality, and fairness of a relationship coffee model is crucial to investigate how power is transmitted in the proximity dimensions and by whom. As power is transversal in our framework, we can analyse for the other dimensions if one party exerts power and can define rules, set standards/prices or determine the relationship more than another party.

### Communication

The third transversal dimension is communication. Reciprocal exchange, feedback, and negotiations are required to build up proximity (Rallet and Torre 1998). Communication is related to other proximity dimensions as it can increase them, but a certain level of cognitive proximity is also a prerequisite for effective communication (Boschma 2005). Virtual and online communication technologies facilitate regular communication over longer timespans and large geographical distances if actors cannot meet face‐to‐face (Dubois 2018).

MacGregor *et al. *(2017) show that direct communication with coffee producers is a principle which all observed leading direct trade firms use. It can happen during personal visits but also via e‐mails or phone conversations. Direct communication in relationship coffee models is supposed to support knowledge exchange, build a common understanding and ‘partnerships that go beyond mere market transactions’ (Borrella *et al.* 2015, p. 34).

## Methods

While the focus of this publication lies in the introduction of the framework, we also want to illustrate its applicability. To test the framework, we used a casestudy, as it is considered as a suitable methodological approach to discover potential ‘gaps and holes’ in theories and frameworks (Ridder 2017). Yin (2009, p. 13) explains that case study research can answer ‘how’ and ‘why’ questions to understand contemporary real‐life phenomena. Purposeful sampling with the use of case study selection criteria assisted in selecting an information‐rich case (Patton 2002) that should be illustrative for relationship coffee models, and that allowed us to analyse all dimensions of the proximity framework elaborated above.

Thus, casestudy selection was based the following criteria: i) a coffee value chain with direct interactions between the value chain actors, ii) a Colombian coffee chain as the national coffee context was already known from previous studies, iii) access to all interviewees, iv) willingness of the interviewees to talk about their relationship openly, and – for this publication – v) simple structure of the value chain to be able to explain the framework properly. The data were gathered in explorative fieldwork in Colombia in November and December 2018. As a first step, cafés and restaurants stating to procure coffee directly from coffee producers were identified and contacted via phone. During the explorative fieldwork, we conducted further interviews to understand various types of relationship coffee models.

In this case, the restaurant owner was contacted, and he then established contact with the coffee producer. Thus, the most important source of information to study proximity in our casestudy were two semi‐structured qualitative interviews with the involved value chain actors (coffee producer and restaurant owner) following interview guidelines. The interviews were in German (the restaurant owner is German, living in Colombia) and Spanish (with the coffee producer). The interviewer ensured a conversational and open discussion and made sure that the information and experiences shared would not negatively impact the interviewees’ trade relationship. The interviews were complemented with observations of the interviewees’ interaction (e.g., phone calls, direct communication among them) and further empirical material (quality reports, coffee packages, photographs).

We triangulated all sources of data and used case study protocols to improve validity and reliability of the casestudy research (see Yin 2009). The interviews were recorded and transcribed and analysed in German and Spanish. We used qualitative coding supported by Atlas.ti software to analyse the collected data. The codes consisted of the proximity dimensions (see Table 1) supplemented with inductive codes that emerged from the case itself. After the fieldwork, we conducted follow‐up conversations via phone and e‐mail with the interviewees to clarify open questions and follow the development of their relationship.

## Illustrative case study to empirically test the analytic proximity framework

**Figure 1:**
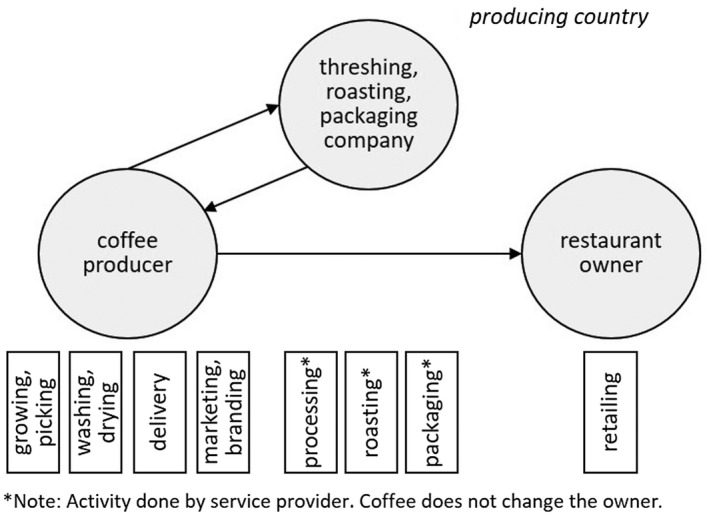
Relationship coffee model of the coffee producer and the restaurant owner

In this section, we describe the casestudy results from one illustrative relationship coffee model in Colombia, which helped to empirically test the proximity framework. The value chain analysed consists of two actors (a coffee producer and a restaurant owner), both located within the same region close to Medellin, a big city in Colombia. In Colombia, around 563,000 families economically depend on coffee production (Quiñones‐Ruiz *et al.* 2015; Andrade and Zapata 2019). While the vast majority of the production is exported, domestic consumption is rising (USDA 2018). According to the interviewees, the number of coffee shops in Medellin has been rising strongly over the last five years, and an increasing number of coffee producers is offering their roasted coffee directly to these coffee shops, restaurants and hotels. According to the interviewees, there is more supply than demand for coffee on the local market, which puts the buyers in advantageous bargaining positions. The coffee producer in our casestudy owns a finca where he cultivates coffee on 16 hectares together with his siblings close to Medellin. At the finca lives an administrator with his family who manages the daily business at the finca as the owner has a fulltime job in the city and economically does not (totally) depend on the coffee production. The producer directly sells 10–15 per cent of the coffee to cafés, hotels, restaurants, and the rest to a cooperative. The producer brings parts of his parchment coffee to a threshing, roasting and packaging company in the city, and then sells the roasted coffee directly to gastronomy (see Figure 1). The restaurant owner, a German living in Colombia, bought the roasted coffee only from this coffee producer who delivered it every week or every second week. The restaurant served the coffee as a drink but also sold the producer’s coffee package with his brand.

Table 3 summarises the proximity dimensions of the coffee producer and the restaurant owner. In this case, the geographical proximity is high (as the actors live in the same region), they met very regularly (every week or every second week). Their communication was direct and regular and no formal contract existed. During the interview, the restaurant owner stated this very clearly: ‘There is no contract. I call and order. And when I am not satisfied anymore, I do not call and order anymore’. Albeit the coffee producer got a higher price for his coffee compared to selling it as parchment coffee to the cooperative, he took all risks of production, processing, delivery up to the restaurant and even possible changes. Consistently high coffee quality was very important for both of them and laid the basis for their relationship, as they did not sign a contract. Both have a relatively high level of overlapping knowledge, but the producer’s knowledge is bigger in the area of coffee growing and production. While the bargaining power between the two actors seemed balanced during the fieldwork, the demonstrative power of the restaurant owner was higher due to an increasing number of coffee producers that offer their roasted coffee to the restaurant.

**Table 3:** Proximity dimensions in the illustrative case study

Proximity dimensions	Subdimensions	Illustrative casestudy: coffee producer – restaurant owner	(Temporary) geographical proximity	Power	Communication
Organisational proximity	Organisational agreements	No formal contracts, orders were done short‐term via telephone	Meet face‐to‐face every 1‐2 weeks, producer comes to the restaurant to deliver the coffee, restaurant owner has not been to the finca	Producer responsible for the coffee up to delivery, can directly negotiate price, but relationship can be ended any time (no formal contract); balanced bargaining power, but producer takes most of the risk, higher demonstrative power of restaurant owner due to an increasing number of coffee producers offering their roasted coffee to the restaurant	Direct/regular communication – producer and restaurant owner used to meet and talk regularly in person and via telephone before the break of the relationship
	Mode of price setting/ negotiation	Direct negotiations between restaurant owner and the coffee producer			
	Duration of trade relations	Regularly, trade for 2 years; after our fieldwork there was a six‐month break			
	Benefit sharing	Producer gets 14.000 COP/pound (= 4,3 USD)			
	Risk sharing	Producer is responsible until delivery			
Institutional proximity	Coffee quality norms/rules/ conventions	Overlapping high quality norms of both actors, coffee has around 85 SCA points, the coffee producer is very rigid about his cup quality as well: gives his coffee to a laboratory which analyses the physical, chemical and in‐cup quality, shared report with restaurant owner			
	Certifications	No certifications			
	Environmental practices	Water‐saving processes important for producer, not particularly important for the restaurant owner			
	Social practices (excl. pay)	No social engagement of restaurant owner to improve producer’s situation			
Cognitive proximity	Knowledge bases of actors	Overlapping knowledge bases, but producer has a bigger knowledge of coffee production process, both have approx. same level of coffee tasting skills			
	Knowledge exchange, learning	Producer provides barista training for the restaurant employees to learn about coffee preparation and to secure a good cup quality			
Social proximity	Trust	No formal contract: both trust each other to deliver quality and quantity and to pay correctly, based on past experiences; however, the restaurant owner suddenly terminated and re‐started the relationship			
	Knowledge about production and each other	Restaurant owner knows basic facts about producer and his finca (region, location), producer has enough knowledge about the restaurant owner to manage the business, but not more than that			
	Degree of personal acquaintance	Business partners (not friends or family)			

However, after the fieldwork, the producer told us that the restaurant owner stopped ordering coffee without further explanation. After a six‐month break, the restaurant owner started occasionally buying small quantities again because his new supplier is currently unable to deliver. When we asked the restaurant owner, why he stopped, he vaguely answered that smaller producers generally make mistakes that lead to lower coffee quality and that he can profit from other producers that provide additional services (e.g., maintenance of coffee machines).

## Discussion

The main objective of this article is to propose an analytic framework to study proximity of actors along coffee value chains. Among others, we draw on Boschma (2005) and proximity literature (Rallet and Torre 1998; Torre and Rallet 2005; Torre 2008; Balland *et al.* 2015; Dubois 2018) and connect it to relationship coffee literature (Borrella *et al.* 2015; Holland *et al.* 2016; Fischer 2017; MacGregor *et al.* 2017; Boaventura *et al.* 2018; Hernandez‐Aguilera *et al.* 2018; Vicol *et al.* 2018). There is no clear definition among roasters what relationship coffee should be (MacGregor *et al.* 2017). Relationship coffees – that include in our definition direct trade coffees – generally emphasise a direct and close relationship within the value chain (Vicol *et al.* 2018). Our framework should enable the differentiation between proximity dimensions. Therefore, it can be a tool to compare different relationship coffee models, but also relationship coffee with conventional coffee value chains. Fischer (2017) shows how roasters and retailers construct value and taste in specialty coffee and precisely concludes that smallholder coffee producers are not the ones benefitting from the high prices in the sector: ‘smallholding farmers control terroir in the gritty materiality of dirt and land, and thus the ability to produce a certain quality, but the real economic power lies with those who define quality, the coffee aficionados and Third Wave marketeers who orchestrate the symbolic and social means of producing’ (Fischer 2017, p. 2). Empirical analysis based on the proximity framework can help to understand the conditions that reproduce underlying power inequities in the coffee value chains (Grabs and Ponte 2019). It adds additional dimensions other than quality and a higher price for green coffee beans – as regularly presented by relationship coffee roasters (see MacGregor *et al.* 2017) – when investigating the well‐meant social and ecological concerns of relationship coffee roasters and retailers. We have to look closer into dimensions like mechanisms of benefit and risk sharing (organisational proximity), practices and norms for social and ecological welfare (institutional proximity), knowledge exchange and learning (cognitive proximity), and personal relationships of the actors (social proximity). When looking at who explicitly or implicitly exerts control over what subjects, definitions, and agenda‐setting, we get a clearer picture of the inherent power structures. By using the framework, it could be possible to investigate if the roasters’ and retailers’ marketed benefits of their relationship coffee model is more than just another coffee attribute like taste, origin or altitude now complemented with the picture of a producer.

The illustrative case study empirically tests the analytic framework. The coffee producer sold his roasted and packaged coffee beans directly to the restaurant owner. Apart from low social proximity, the case shows high geographical, institutional and cognitive proximity. Both actors based their valuation of the trade’s success primarily on a high and consistent cup quality. Both seemed to have an overlapping knowledge base in terms of coffee tasting and preparation and negotiated price, quality, and quantity directly with each other. The coffee producer received a relatively higher price for his beans (approx. 4,30 USD/pound roasted beans) compared to the median FOB price for a Colombian 85 points green specialty coffee (+/− 3 USD) (Transparent Trade Coffee 2018). However, the coffee producer not only bore the risks and costs of coffee production but also of processing, roasting, packaging, and distribution. A balanced bargaining power was offset with imbalances in the demonstrative power due to an increasing number of coffee producers that offer their coffee at the restaurant.

While 75 per cent of Colombian coffee beans are exported (Panhuysen and Pierrot 2018) – the coffee in our case is grown, roasted and consumed in the same region. The producer presented here works in a fulltime job outside the coffee sector, coffee is just a side business and he does not depend on living from it. Therefore, it is easier for him to access knowledge beyond coffee production, and to take (production) decisions that involve higher risk. Even though both actors stressed that the trust in their business relationship is based on past experiences of good collaboration, the social proximity dimension is the weakest. This together with the power imbalance due to a high number of coffee producers engaging in selling roasted coffee might explain why the restaurant owner so easily terminated the relationship.

Generally speaking, too little cognitive proximity (very different knowledge bases) of the actors in the coffee business traditionally created inequality: while producers mainly control material quality of coffee beans, roasters and retailers usually have power over symbolic and in‐person service quality, and can obtain a larger share of the pie (Daviron and Ponte 2005). Quality is defined by large buyers in the global North (Grabs and Ponte 2019). In theory, heterogeneous knowledge bases (low cognitive proximity) can become the starting point for mutual learning processes (Balland *et al.* 2015). As relationship coffee models like other AFNs are not static (Jarosz 2008), coffee producers could learn to derive value also from symbolic or even in‐person service quality. In the past years, the third wave of coffee also spilt over to cities in coffee producing countries, leading to higher geographical proximity of coffee producers and roasters there (FNC 2013; Pongsiri 2013; Noppakoonwong *et al.* 2014; Vicol *et al.* 2018; Purnomo *et al.* 2019). Also some initiatives have been established that import coffee roasted at origin (e.g., Moyee, Kaffee Kooperative, and Moema). It would be interesting to see how power, proximity, and learning are influenced by and will influence these developments.

In the coffee sector, national regulations and contexts strongly influence production and trade (e.g., Ponte 2002; Daviron and Ponte 2005; Barjolle *et al.* 2017). However, as relationship coffee buyers often offer coffee from several origins and producers, the framework makes it possible to compare these chains – also if producers and buyers are located in different countries. This requires careful contextualisation of the specific value chain during the analysis.

A potential limitation in the framework application is related to methodical issues: when the data are gathered through qualitative semi‐structured interviews, it is crucial i) to talk to all actors involved in the value chain to get a balanced picture and ii) to establish an open interview atmosphere to encourage interviewees to share their – partially sensitive – experiences. As our experience shows, the case‐study only depicts the relationship in a certain point in time. The evolution of the relationship is harder to grasp. As the illustrated value chain is very short, research on more complex value chains might include further data (e.g., contracts, maps, charts, lists, survey data, etc.) and (participant) observation to get a richer picture of proximity.

Further comparative analysis based on our framework could help to distinguish different types of relationship coffee models. An extension of the research with quantitative data to quantify the interactions between the dimensions (e.g., the influence of geographical proximity on the other dimensions) or performance measures could deliver valuable results. More research is also needed to test the applicability of the framework to other agro‐food products like cotton or cacao with similar value chain patterns (Gibbon 2001, Staritz *et al*. 2017). Another field of application could be AFNs with direct interaction of actors from production and consumption. Drawing on the findings of Dubois (2018), it would be insightful to analyse AFNs with our analytic framework to further understand their proximities and implications for the actors of the value chain.

## Conclusion

Current challenges in conventional coffee value chains prevail. While certifications like Organic or Fairtrade were trying to improve social and ecological challenges, criticism persists. Relationship coffee models emerged to outperform these certifications, to acquire high‐quality coffee and to enhance the situation in coffee producing communities. However, the model and terms (relationship coffee, direct trade coffee) have been co‐opted by firms that adhere less to the high moral standards of the relationship coffee pioneers. Thus, in this article, we develop a framework to analyse the proximity between the actors of relationship coffee models. With our framework we aim to provide the opportunity to make proximities and distances in the concerned dimensions visible and to better understand the implications of different dimensions of proximity for the interactions between producers and buyers/roasters. Additionally, the proposed framework aims to unveil the conditions supporting or impeding producers in relationship coffees models. Our illustrative case to test the framework looks at the relationship between a coffee producer and a restaurant owner. They are both located in the same region in a coffee producing country. Despite high levels of geographical, organisational, institutional and cognitive proximity, the low social proximity and unequal power due to a high number of coffee producers that sell roasted coffee showed the vulnerability of business relations in a relationship coffee model.

Relationship coffee literature (i.e., Borrella *et al.* 2015; Boaventura *et al.* 2018; Hernandez‐Aguilera *et al.* 2018; Vicol *et al.* 2018) calls for more research to open the black box of relationship coffee models. We propose and tested a proximity framework that can support comparative research on different types of relationship coffees, as well as on other value chains and AFNs. Comparative research on effects, opportunities and potential pitfalls of relationship coffee models is much needed to gather knowledge on how to design fairer value chains.

## Conflict of interest statement

There is no conflict of interest to declare.

## Data Availability

The data that support the findings of this study are available on request from the corresponding author. The data are not publicly available due to privacy restrictions.
